# Psychological Distress, Early Behavioral Response, and Perception Toward the COVID-19 Pandemic Among Health Care Workers in North Shoa Zone, Oromiya Region

**DOI:** 10.3389/fpsyt.2021.628898

**Published:** 2021-05-13

**Authors:** Kemal Jemal, Berhanu Senbeta Deriba, Tinsae Abeya Geleta

**Affiliations:** ^1^Department of Nursing, College of Health Sciences, Salale University, Fitche, Ethiopia; ^2^Department of Public Health, College of Health Sciences, Salale University, Fitche, Ethiopia

**Keywords:** COVID-19, psychological distress, depression, anxiety, insomnia, perception, behavioral response, health care workers

## Abstract

**Background:** The coronavirus disease 2019 (COVID-19) pandemic has had a significant psychological impact on health care workers (HCWs). Therefore, this study inspects the mental health status, behavioral response, and perception among HCWs (nurses, physicians, and medical laboratory workers) during the COVID-19 pandemic in public health care facilities.

**Methods:** A facilities-based cross-sectional study was conducted in July 2020. A simple random sampling technique was used to select study participants. Data were collected by self-report administered questionnaires using Patient Health Questionnaire-9 (PHQ-9) for depression, General Anxiety Disorder-7 (GAD-7) for anxiety, Insomnia Severity Index (ISI) for insomnia, Impact of Event Scale-Revised (IES-R) for psychological distress, Perceived Threat Scale for perception, and Behavioral Response Inquiry for the behavioral response. Moreover, bivariable and multivariable logistic regressions analysis was used to identify the association between dependent and independent variables at *p*-value <0.05.

**Results:** A total of 417 (98.6%) HCWs responded to a self-administered questionnaire. The proportion of HCWs who had moderate to severe symptoms of psychological distress, depression, anxiety, and insomnia during the COVID-19 pandemic were 58, 16.3, 30.7, and 15.9%, respectively. Three-fifth of the nurses, medical laboratory professionals (62.2%), and physicians (59.2%) had reported good behavioral responses toward the COVID-19 pandemic. More than three-fifths of the nurses had reported poor perception toward the COVID-19 pandemic. Conversely, 61.2% of physicians and three-fourths (75.5%) of medical laboratory professionals had reported good perception toward the COVID-19 pandemic. Female and married participants, those working in the emergency unit, those with poor behavioral responses, and those with poor perception toward the COVID-19 pandemic were significantly associated with symptoms of psychological distress, depression, anxiety, and insomnia.

**Conclusions:** Psychological impacts among physicians, nurses, and medical laboratory professionals are high during the COVID-19 pandemic. The Ethiopian Federal Ministry of Health should aim to protect all HCWs' psychological well-being during the COVID-19 pandemic with appropriate interventions and accurate information response.

## Introduction

The outbreak of COVID-19 was reported in Wuhan in December 2019, from where it rapidly spread within Wuhan and then worldwide (1). The coronavirus is a new type of virus that did not exist before in humans. It causes common respiratory symptoms, such as fever, cough, shortness of breath, and other respiratory problems. In more severe cases, the infection can cause pneumonia, severe acute respiratory syndrome, kidney failure, and even death (2–4).

As of 31 July 2020, the pandemic had caused more than six million infections, 94.26% of which recovered, with death rates of 5.74% worldwide. In Africa and sub-Saharan regions, more than 100,000 infected cases and 4,000 deaths have occurred. Ethiopia is one of the sub-Saharan countries affected by COVID-19, with more than 17,530 cases and 274 deaths reported (5). These high severe morbidities and mortalities of the ongoing COVID-19 pandemic cause depression, anxiety, insomnia, and psychological distress among HCWs (6). Furthermore, HCWs have experienced depression, devastating health care facilities or working areas, fear of feeling sick or dying themselves, feelings of helplessness, lack of sleep, self-harm or suicide, and stigma during the COVID-19 pandemic (7).

A study conducted in New York, USA reported that 57% of HCWs had reported acute distress, 48% had depression, 33% had anxiety, and 26% had severe insomnia (8). A study conducted in China found that the symptoms of anxiety, depression, insomnia, and psychological distress were present in 44.6, 50.4, 34.0, and 71.5% of HCWs, respectively (9). Another study conducted in China showed that 73.4% of HCWs on the front line in the hospital suffered from extreme distress levels, 50.7% had depressive symptoms, 44.7% had anxiety symptoms, and 36.1% had insomnia (10). Similarly, a study conducted in Italy revealed that 24.7% of HCWs had depression symptoms, 19.8% had anxiety symptoms, 8.27% had insomnia, and 21.9% had perceived stress symptoms (11). Additionally, Turkey's result showed that 77.6% of HCWs had depression symptoms, 60.2% had anxiety symptoms, 50.4% had insomnia, and 76.4% had psychological distress symptoms (12).

Knowing health care perception and early behavioral responses is very important for developing a coping strategy targeted to fight the COVID-19 pandemic and protect HCWs from mental distress. It is highly problem-focused, and most people listen to experts' advice and try to behave calmly and appropriately by accepting measures to tackle COVID-19 (13). Early behavioral responses of the HCWs play an essential role in the control of the outbreak. Previous studies have explored this topic in various cultural settings with COVID-19 behavioral responses (14). Behavioral responses are also associated with government involvement level, perceptions of diseases, and the outbreak stage, which varies by disease severity and level of settings (15, 16).

The Ethiopian government is controlling the COVID-19 pandemic through encouraging social distancing and school closures, deploying new medical staff to healthcare institutions, running intense public messaging campaigns through the media, and research activities. Despite many efforts to minimize the COVID-19 pandemic, the newly emerging disease's psychological effects among HCWs remain unaddressed. Therefore, we aimed to assess the prevalence of psychological distress, depression, anxiety, insomnia, early behavior response, and perception toward the COVID-19 pandemic associated with health care providers in North Shoa health care facilities.

## Methods

### Study Area and Period

The study was conducted in four hospitals and 16 health centers in July 2020 in the North Shoa Zone of the Oromia region. The North Shoa zone is one of 20 zones found in the Oromia regional state. Fitche is a zonal administrative town located 114 kilometers from the country's capital city, Addis Ababa. The North Shoa Zone has two general hospitals and two district hospitals, 63 health centers, 268 health posts, and more than 2,000 health professionals.

### Study Design and Participants

A facility-based cross-sectional study was implemented. The study sample size was determined using a single population proportion formula, assuming a 50% proportion, since no study was conducted earlier. Considering a 95% confidence interval and a 5% marginal error with a 10% non-response rate, the final sample size was 423 HCWs. The HCWs working in the North Shoa Zone public health-care facilities were included in the study and those working in private health care facilities and on annual leave were excluded from the study. The proportional allocation was done to each health care facility, depending on their HCWs. The study participants were selected using a simple random sampling technique from each department list (emergency, surgical, medical, COVID-19 treatment center, medical laboratory, and outpatient department).

### Data Collection Tools and Procedure

Self-administered data were collected by four BSc psychiatry nurses and supervised by two MSc psychiatry health professionals. Three days of training were provided for data collectors and supervisors on how to maintain the purpose of the study, details of the questionnaire, the importance of privacy, and ensuring the respondents' confidentiality. In addition to data collection training, the prevention technique of the COVID-19 pandemic with personal protective equipment was provided for data collectors and supervisors. Facemasks and sanitizers were also provided for HCWs who were participated in self-administered questionnaires.

The questionnaire was adapted from different literature (12, 17, 18) that was initially written in the English language, translated to Afan Oromo and Amharic, and then back-translated to English by language experts to check its consistency. The questionnaire collected a variety of information: socio-demographic characteristic, working department, use of personal protective equipment, experienced symptoms (cough and fever) during the past 2 weeks, confirmed or suspected cases of COVID-19 pandemic infections patients in their health care setting, and friends or relatives with symptoms in the past 2 weeks. Participants were asked whether they had used the online training platform on COVID-19 infection prevention and control methods, provided by the Ethiopian Ministry of Health website (19).

Depression was measured using PHQ-9, which contains a nine-item self-report scale used to screen for symptoms of depression, ranging from 0 to 27. Scores were categorized as normal (0–4), mild (5–9), moderate (10–14), or severe (15 and above) (20). Anxiety was measured using GAD-7, which contains a seven-item self-reported questionnaire planned to screen the symptoms of anxiety. It is categorized as normal (0–4), mild (5–9), moderate 10–14), and severe (15 and above) (21). Insomnia was measured using ISI, which is a seven-item self-report questionnaire evaluating the nature, severity, and impact of insomnia. The scores range from normal (0–7), sub-threshold (8–14), moderate (15–21), to severe (22 and above) (22). The psychological distress of COVID-19 was measured using the IES-R, which contains 22-items. The total IES-R score was divided into normal (0–8), mild (9–25), moderate (26–43), and severe (44 and above) (23).

Participants were asked 11 Likert scale questions for behavior response about recent avoidance behaviors (avoided eating out, avoided taking public transportation, reduced visits to public places, increased surface cleaning, and maintained better indoor ventilation) in response to the pandemic (24–26). The perceptions were measured with nine items questioning whether HCWs believed that specific measures would reduce their risk of catching COVID-19, with possible responses measured on a five-point Likert scale with options from very unlikely (1) to very likely (5) (13, 27).

The translated Afan Oromo and Amharic versions of data collection tools were pre-tested at Shano Primary Hospital among a sample of 21 (5%) HCWs. The questionnaire was checked for completeness and inconsistency with the original one by the supervisor. Accordingly, after discussion with data collectors and supervisors, a necessary measure was taken, and modification was done before actual data collection. Again, in the pre-tested questionnaire, the reliability of each subscale was done, with each item attaining a Cronbach's alpha of 0.827 for psychological distress, 0.672 for depression, 0.718 for anxiety, 0.856 for insomnia, 0.595 for early behavior response, and 0.643 for perception.

### Data Processing and Analysis

The collected data were entered into Epi-data version 4.2.3 and transferred to SPSS version 23 for further analysis (coded, edited, and cleaned). The descriptive data analysis was done and presented in frequency and percentage. The outcome variables were dichotomized into normal or depressed, normal or anxious, normal or insomnia, and normal or psychological distress depending on the cut-off point ≥5 for PHQ-9, ≥5 for GAD-7, ≥8 for ISI, and ≥9 for IES-R, respectively. Bivariate and multivariate logistic regression analyses were carried out, and variables with a *p*-value ≤ 0.2 in the bivariate analysis were included in a multivariable logistic regression analysis to control the confounding effect variables (28). Multiple logistic regressions were done using backward stepwise methods to identify factors associated with psychological distress, depression, anxiety, and insomnia. The model goodness-of-fit test was checked by Hosmer–Lemeshow goodness of fit, and the *p*-value of the model fitness test was 0.87 for psychological distress, 0.75 for depression, 0.69 for anxiety, and 0.84 for insomnia. Finally, adjusted odds ratio (AOR) with 95% confidence interval (CI), and *p*-value < 0.05 were considered statistically significant.

### Ethical Consideration

Ethical clearance was obtained from the Salale University Ethical Review Committee, and the study protocol and methodology were approved on June 15, 2020, with approval number SLUERC/046/2020. After thoroughly discussing the study's ultimate purpose and method, written consent was sought from the North Shoa Zonal health bureau/woreda department, and an informed written consent form was obtained from each respondent. The confidentiality of respondents was ensured by excluding their names from the questionnaire and kept in a password-locked computer.

## Resuts

We have distributed the survey to 423 HCWs and received a response from 417 (98.6%) participants. The majority of the respondents were male (66.9%), aged 20–30 years (41.2%), married (58%), bachelor's degree educated (40.5%), nursing professionals (29%), and had 5–10 years of working experience (40.8%). More than one-fifth (23.3%) of HCWs had worked in the COVID-19 treatment center. Of them, 24.9, 13.3, and 29.6% were nurses, physicians, and medical laboratory professionals, respectively (Table 1).

**Table 1:** Socio-demographic and COVID-19-related characteristics of health-care workers in North Shoa Zone, Oromiya region July 2020 (*n* = 417).

**Variables**	**No. (%)**			
	**Total**	**Health care workers**		
		**Nurses**	**Physicians**	**Medical laboratory**
Overall	417 (100)	221 (53.0)	98 (23.5)	98 (23.5)
**Age**				
20–30	172 (41.2)	74 (33.5)	44 (44.9)	54 (55.1)
30–40	155 (37.2)	103 (46.6)	20 (20.4)	32 (32.7)
≥40	94 (21.6)	44 (19.9)	34 (34.7)	12 (12.2)
**Sex**				
Male	279 (66.9)	141 (63.8)	82 (83.7)	56 (57.1)
Female	138 (33.1)	80 (36.2)	16 (16.3)	42 (42.9)
**Marital status**				
Single	175 (42.0)	101 (45.7)	42 (42.9)	32 (32.7)
Married	242 (58.0)	120 (54.3)	56 (57.1)	66 (67.3)
**Educational status**				
Diploma	140 (33.6)	108 (48.9)	0 (0)	32 (32.7)
BSc/MD	177 (42.4)	55 (24.9)	56 (57.1)	66 (67.3)
MSc/specialty	100 (24.0)	58 (26.2)	42 (42.9)	0 (0)
**Working unit/department**				
Emergency	77 (18.5)	56 (25.3)	21 (21.4)	0 (0)
Surgical	56 (13.4)	30 (13.6)	26 (26.5)	0 (0)
Medical	55 (13.2)	27 (12.2)	28 (28.6)	0 (0)
COVID-19 treatment center	97 (23.3)	55 (24.9)	13 (13.3)	29 (29.6)
Medical laboratory	69 (16.5)	0 (0)	0 (0)	69 (70.4)
Outpatient department	63 (15.1)	53 (24.0)	10 (10.2)	0 (0)
**Year of experience**				
<5 year	141 (33.8)	77 (34.8)	36 (36.7)	28 (28.6)
5–10 years	170 (40.8)	100 (45.3)	36 (36.7)	34 (34.7)
>10 years	106 (25.4)	44 (19.9)	26 (26.6)	36 (36.7)
**Taking online training about COVID-19**				
No	136 (32.6)	78 (35.3)	38 (38.8)	20 (20.4)
Yes	281 (67.4)	143 (64.7)	60 (61.2)	78 (79.6)
**Availability of personal protective equipment**				
No	299 (71.7)	159 (71.9)	74 (75.5)	66 (67.3)
Yes	118 (28.3)	62 (28.1)	24 (24.5)	32 (32.7)
**Experience symptom of COVID-19**				
No	214 (51.3)	97 (43.9)	59 (60.2)	58 (59.2)
Yes	203 (48.7)	124 (56.1)	39 (39.8)	40 (40.8)
**Friend or families experience a symptom of COVID-19**				
No	327 (78.4)	167 (75.6)	83 (84.7)	77 (78.6)
Yes	90 (21.6)	54 (24.4)	15 (15.3)	21 (21.4)
**Noticed the COVID-19 cases in your institution**				
No	243 (58.3)	106 (48)	76 (77.6)	61 (62.2)
Yes	174 (41.7)	115 (52)	22 (22.4)	37 (37.8)

More than two-thirds (67.4%) of HCWs had trained online about COVID-19 infection prevention and control, and 71.7% had a shortage of personal protective equipment. One-fourth of nurse's friends or families had experienced symptoms of COVID-19, followed by medical laboratory professionals (921.4%) and physicians (15.3%). More than half of nurses had experienced COVID-19-like symptoms during the COVID-19 pandemic (56.1%), followed by medical laboratory professionals (40.8%) and physicians (39.8%). Nearly half of nurses had observed COVID-19 cases in their working institution (52.0%), whereas 22.4% of physicians and 37.8% of medical laboratory professionals had observed the COVID-19 in their working institution (Table 1).

Of the HCWs, 158 (37.9%) had received and read sufficient information regarding the COVID-19 pandemic, and 160 (38.4%) of the study participants had self-perceived severity about the COVID-19 pandemic. More than one-fourth (26.9%) of HCWs were confused about the information reliability of the COVID-19, and 28.3% of respondents believed they had contracted COVID-19 during the current outbreak (Table 2).

**Table 2:** Perception toward COVID-19 pandemic among HCWs in North Shoa Zone health care facilities, Oromiya region, July 2020 (*n* = 417).

**Perception**	**Very unlikely**	**Unlikely**	**Moderately**	**Likely**	**Very likely**
	***N* (%)**	***N* (%)**	***N* (%)**	***N* (%)**	***N* (%)**
Self-perceived risk of COVID-19	42 (10.1)	88 (21.1)	166 (39.8)	58 (13.9)	63 (15.1)
Self-perceived severity if contracted COVID-19	40 (9.6)	54 (12.9)	160 (38.4)	107 (25.7)	56 (13.4)
Received and read sufficient information brochures about COVID-19	39 (9.4)	46 (11.0)	158 (37.9)	94 (22.5)	80 (19.2)
Confused about information reliability about COVID-19	56 (13.4)	107 (25.7)	94 (22.5)	112 (26.9)	48 (11.5)
Confidence in taking measures to protect me and others from COVID-19	22 (5.3)	101 (24.2)	136 (32.6)	58 (13.9)	100 (24.0)
Confidence in own doctor's ability to diagnose or recognize	52 (12.5)	56 (13.4)	179 (42.9)	80 (19.2)	50 (12.0)
Contracting COVID-19 during the current outbreak	66 (15.8)	81 (19.4)	96 (23.0)	118 (28.3)	56 (13.4)
Surviving if infected with COVID-19	44 (10.6)	40 (9.6)	160 (38.4)	89 (21.3)	84 (20.1)
Concerns about other family members getting COVID-19 infection	42 (10.1)	100 (24.0)	93 (22.3)	90 (21.6)	92 (22.1)
Minimum to Maximum				14–41	
Mean ±SD				28.40 ± 6.397	

More than half (51.8%) of the study participants had a good perception of COVID-19 infection. More than three-fifths (62.9%) of the nurses had reported poor perception toward the COVID-19 pandemic. Conversely, 61.2% of physicians and three-fourths (75.5%) of medical laboratory professionals had reported good perception toward the COVID-19 pandemic (Table 4).

More than half (53.5%) of the study participants wore masks and 35.3% had washed their hands with soap and water more often than usual. One hundred seventy-two (41.2%) HCWs have always covered their mouths when coughing and sneezing. During the current COVID-19 pandemic, 142 (34.1%) health care facilities increased surface cleaning, and 122 (29.3%) had constantly maintained better indoor ventilation in the healthcare facilities (Table 3).

**Table 3:** Early behavioral response of HCWs to COVID-19 pandemic in North Shoa Zone health care facilities, Oromiya region, July 2020 (*n* = 417).

**Behavioral response to COVID-19**	**Never**	**Occasionally**	**Sometime**	**Most of the time**	**Always**
	***N* (%)**	***N* (%)**	***N* (%)**	***N* (%)**	***N* (%)**
Covering mouth when coughing and sneezing	32 (7.7)	32 (7.7)	58 (13.9)	123 (29.5)	172 (41.2)
Avoiding sharing of utensils during meals	34 (8.2)	42 (10.1)	150 (36.0)	81 (19.4)	110 (26.4)
Washing hands with soap and water more often than usual	18 (4.3)	40 (9.6)	66 (15.8)	110 (26.4)	183 (43.9)
Washing hands immediately after, coughing, rubbing the nose, or sneezing	14 (3.4)	40 (9.6)	152 (36.5)	64 (15.3)	147 (35.3)
Washing hands after touching contaminated objects and patient	10 (2.4)	18 (4.3)	94 (22.5)	100 (24.0)	195 (46.8)
Wearing mask regardless of the presence or absence of symptoms	16 (3.8)	24 (5.8)	78 (18.7)	76 (18.2)	223 (53.5)
Avoid eating outside in a café or hotel	18 (4.3)	50 (12.0)	176 (42.2)	59 (14.1)	114 (27.3)
Avoid public places/events	18 (4.3)	56 (13.4)	112 (26.9)	93 (22.3)	138 (33.1)
Avoiding public transport (subway, tram, bus, train).	44 (10.6)	64 (15.3)	101 (24.2)	78 (18.7)	130 (31.2)
Maintaining better indoor ventilation in health-care	28 (6.7)	74 (17.7)	90 (21.6)	103 (24.7)	122 (29.3)
Increasing surface cleaning in health care	22 (5.3)	22 (5.3)	101 (24.2)	130 (31.2)	142 (34.1)
Minimum to Maximum				11–55	
Mean ±SD				41.15 ± 9.638	

Overall good early behavioral responses to the COVID-19 pandemic were reported in 253 (60.7%) of the HCWs (nursing, physicians, and laboratory professionals). More than three-fifths of the medical laboratory professionals (62.2%) and nurses (60.6%) had reported good early behavioral responses toward the COVID-19 pandemic, followed by physicians (59.2%) (Table 4).

**Table 4:** The prevalence of psychological distress, depression, anxiety, and insomnia during the COVID-19 pandemic among HCWs in North Shoa Zone health care facilities, Oromiya region, July 2020 (*n* = 417).

**Variables**	**No. (%)**			
	**Total**	**Health care workers**		
		**Nurses**	**Physicians**	**Medical laboratory**
Overall	417 (100)	221 (53.0)	98 (23.5)	98 (23.5)
**Psychological distress (IES-R)**				
Normal	120 (28.8)	87 (39.4)	21 (21.5)	12 (12.2)
Mild	55 (13.2)	23 (10.4)	16 (16.3)	16 (16.4)
Moderate	46 (11.0)	15 (6.8)	2 (2.0)	29 (29.6)
Severe	196 (47.0)	96 (43.4)	59 (60.2)	41 (41.8)
**Depression (PHQ-9)**				
Normal	291 (69.8)	156 (70.6)	77 (78.6)	58 (59.2)
Mild	58 (13.9)	37 (16.8)	6 (6.1)	15 (15.3)
Moderate	48 (11.5)	20 (9.0)	11 (11.2)	17 (17.3)
Severe	20 (4.8)	8 (3.6)	4 (4.1)	8 (8.2)
**Anxiety (GAD-7)**				
Normal	200 (48.0)	111 (50.2)	51 (52.0)	38 (38.8)
Mild	89 (21.4)	52 (23.5)	14 (14.3)	23 (23.5
Moderate	92 (22.0)	43 (19.5)	24 (24.5)	25 (25.5)
Severe	36 (8.6)	15 (6.8)	9 (9.2)	12 (12.2)
**Insomnia (ISI)**				
Normal	299 (71.7)	156 (70.6)	70 (71.4)	73 (74.5)
Mild	52 (12.5)	35 (15.8)	13 (13.3)	4 (4.1)
Moderate	42 (10.2)	19 (8.6)	14 (14.3)	9 (9.2)
Severe	24 (5.6)	11 (5.0)	1 (1.0)	12 (12.2)
**Perception against COVID-19**				
Poor perception	201 (48.2)	139 (62.9)	38 (38.8)	24 (24.5)
Good perception	216 (51.8)	82 (37.1)	60 (61.2)	74 (75.5)
**Early behavioral response to COVID-19**				
Poor early behavioral response	164 (39.3)	87 (39.4)	40 (40.8)	37 (37.8)
Good early behavioral response	253 (60.7)	134 (60.6	58 (59.2)	61 (62.2)

### The Prevalence of Psychological Distress, Depression, Anxiety, and Insomnia

The overall prevalence of psychological distress symptoms among HCWs revealed that 13.2% (55) of HCWs had mild symptoms of psychological distress, 11.0% (46) had moderate psychological distress, and 47.0% (196) had severe psychological distress. More than two-thirds (71.4%) of medical laboratory professionals had reported moderate to severe symptoms of psychological distress, followed by physicians (62.2%) and nurses (50.2%) (Table 4).

The overall prevalence of depression symptoms among HCWs during COVID-19 in rural areas indicated that 13.9% (58) of HCWs had mild symptoms of depression, 11.5% (48) had moderate symptoms of depression, and 4.8% (20) had severe symptoms of depression. A high proportion of moderate to severe symptoms of depression during the COVID-19 pandemic was seen among medical laboratory professionals (25.5%), followed by physicians (15.3%) and nurses (12.6%) (Table 4).

There was an overall prevalence of anxiety symptoms among HCWs in the current COVID-19 pandemic. Of the HCWs, 21.3% (89) had mild symptoms of anxiety, 22.1% (92) had moderate symptoms of anxiety, and 8.6% (36) had severe symptoms of anxiety. Similarly, medical laboratory professionals had reported a moderate to severe high proportion of anxiety symptoms (37.7%), followed by physicians (33.7%) and nurses (26.3%) (Table 4).

The overall prevalence of insomnia among HCWs during the current COVID-19 pandemic indicated that 12.5% (52) of HCWs had mild insomnia, 10.1% (42) had moderate insomnia, and 5.8% (24) had severe insomnia. More than one-fifth (21.4%) of medical laboratory professionals reported moderate to severe symptoms of insomnia, followed by physicians (15.3%) and nurses (13.6%) (Table 4).

### Factors Associated With Psychological Distress, Depression, Anxiety, and Insomnia

In the multivariable logistic analysis, female participants were two times more likely to have symptoms of psychological distress than male participants [AOR = 1.89; 95% CI = (1.04, 3.81)]. Married participants were three times more likely to have symptoms of psychological distress than single participants [AOR = 3.09; 95% CI = (2.06, 4.75)]. HCWs who have bachelor's degrees were three times more likely to have symptoms of psychological distress than masters and specialists [AOR = 3.28; 95% CI = (1.49, 6.19)]. Medical laboratory professionals were four times more likely to have symptoms of psychological distress than nursing professionals [AOR = 3.92; 95% CI = (1.92, 4.64)]. HCWs working in the surgical unit were four times more likely to have symptoms of psychological distress than those in the outpatient department [AOR = 3.94; 95% CI = (1.65, 5.30)]. HCWs who have poor perception toward the COVID-19 were three times more likely to have symptoms of psychological distress when compared with HCWs who have a good perception of the COVID-19 pandemic [AOR = 3.27; 95% CI = (1.98, 4.47)] (Table 5).

**Table 5:** Factors associated with psychological distress, depression, anxiety, and insomnia during the COVID-19 pandemic among HCWs in North Shoa Zone health care facilities, Oromiya region, Ethiopia, July 2020 (*n* = 417).

**Variables**	**Psychological impact**		**Depression**		**Anxiety**		**Insomnia**	
	**COR (95% CI)**	**AOR (95% CI)**	**COR (95% CI)**	**AOR (95% CI)**	**COR (95% CI)**	**AOR (95% CI)**	**COR (95% CI)**	**AOR (95% CI)**
**Age**								
20–29	1	1	1	1	1	1	1	1
30–39	1.59 (0.87, 2.91)	1.20 (0.70, 1.82)	0.42 (0.26, 0.69)^*^	0.37 (0.19, 1.74)	1.99 (1.19, 3.35)^*^	1.50 (0.76, 2.97)	0.64 (0.30, 1.35)	0.50 (0.42, 1.32)
≥40	0.52 (0.29, 0.91)^*^	0.35 (0.88, 1.39)	0.73 (0.42, 1.25)	0.51 (0.23, 1.16)	1.35 (0.80, 2.28)	1.59 (0.77, 2.08)	1.18 (0.54, 2.52)	1.06 (0.31, 1.98)
**Sex**								
Male	1	1	1	1	1	1	1	1
Female	3.30 (1.93, 5.62)^*^	1.89 (1.04, 3.81)^*^	2.48 (1.61, 3.84)^*^	1.86 (1.20, 3.56)^*^	2.24 (1.47, 3.42)^*^	1.90 (1.12, 3.24)^*^	2.41 (1.09, 5.33)^*^	2.16 (1.58, 4.38)^*^
**Marital Status**								
Single	1	1	1	1	1	1	1	1
Married	3.46 (2.22, 5.38)^*^	3.09 (2.06, 4.75)^*^	3.57 (2.21, 5.77)^*^	2.87 (2.03, 4.30)^*^	1.99 (0.74, 1.60)^*^	0.99 (0.82, 1.26)	3.65 (1.65, 8.06)^*^	3.31 (1.56, 6.68)^*^
**Education**								
Diploma	2.26 (1.33, 3.84)^*^	2.14 (1.06, 3.80^*^	1.63 (0.90, 2.94)	1.38 (0.59, 2.13)	1.46 (0.87, 2.45)	1.21 (0.59, 2.47)	0.88 (0.39, 1.97)	0.65 (0.67, 1.77)
Bachelor	5.56 (3.15, 9.80)^*^	3.28 (1.49, 6.19)^*^	1.82 (1.03, 3.20)^*^	1.47 (0.66, 2.46)	1.92 (1.17, 3.16)^*^	1.43 (0.49, 2.89)	0.78 (0.36, 1.71)	0.70 (0.21, 1.68)
Masters/specialty	1	1	1		1	1	1	1
**Profession**								
Nurses	1	1	1	1	1	1	1	1
Physicians	2.20 (1.28, 3.80)^*^	2.02 (1.35, 5.56)^*^	0.60 (0.37, 0.99)^*^	0.49 (0.33, 1.45)	0.59 (0.63, 1.02)	0.62 (0.64, 1.29)	0.89 (0.38, 2.10)	0.30 (0.09, 1.05)
Medical laboratory	4.57 (2.36, 8.85)^*^	3.92 (1.92, 6.64)^*^	1.62 (0.21, 0.74)^*^	1.30 (0.64, 1.66)	0.58 (0.33, 1.03)	0.37 (0.18, 1.74)	1.96 (0.97, 3.97)	1.70 (0.82, 1.60)
**Working unit**								
Emergency	2.00 (1.99, 4.03)	1.05 (1.19, 2.50)^*^	2.32 (1.13, 4.79)^*^	2.11 (1.27, 4.61)^*^	2.03 (1.03, 3.99)^*^	2.79 (1.59, 4.89)^*^	4.07 (1.43, 11.58)^*^	2.74 (1.85, 6.45)^*^
Surgical unit	6.67 (2.50, 17.80)^*^	3.94 (1.65, 5.30)^*^	0.98 (0.43, 2.24)	0.91 (0.34, 2.31)	4.16 (1.91, 9.04)^*^	3.26 (2.53, 6.89)^*^	0.66 (0.15, 2.88)	0.61 (0.15, 1.55)
Medical unit	5.49 (2.15, 13.99)^*^	3.14 (1.36, 5.31)^*^	0.73 (0.31, 1.75)	0.70 (0.78, 2.53)	0.52 (0.24, 1.14)	0.39 (0.13, 1.85)	0.91 (0.23, 3.57)	0.56 (0.16, 1.570
COVID-19 unit	1.30 (0.68, 2.47)	0.36 (0.42, 1.39)	0.91 (0.44, 1.91)	0.64 (0.38, 2.53)	2.17 (1.14, 4.14)^*^	2.01 (1.11, 4.01)	0.50 (0.13, 1.93)	0.73 (0.20, 1.98)
laboratory unit	1.96 (0.96, 4.02)	0.52 (0.11, 1.15)	2.01 (0.95, 4.22)	1.28 (0.09, 1.85)	1.66 (0.83, 3.31)	1.50 (0.69, 1.96)	1.52 (0.47, 4.92)	0.38 (0.09, 1.39)
Outpatient unit	1	1	1	1	1	1	1	1
**Experience**								
<5 Year	0.71 (0.39, 1.29)	0.61 (0.31, 1.64)	3.19 (1.77, 5.75)^*^	2.07 (1.89, 4.84)^*^	1.26 (0.75, 2.09)	1.01 (0.54, 1.91	2.51 (1.08, 5.54)^*^	2.45 (1.28, 4.90)^*^
5–10 years	0.48 (0.27, 0.85)^*^	0.37 (0.46, 1.26)	1.60 (0.88, 2.89)	1.05 (0.47, 2.34)	0.64 (0.39, 1.04)	0.56 (0.30, 1.03)	0.93 (0.37, 2.36)	0.81 (0.33, 1.89)
>10 years	1	1	1	1	1	1	1	1
**Online training**								
No	0.47 (0.30, 0.72)^*^	0.41 (0.21, 0.79)^*^	0.76 (0.48, 1.21)	0.62 (0.39, 1.35)	1.50 (0.99, 2.27)	1.42 (0.73, 1.69)	1.85 (0.98, 3.48)	1.21 (0.63, 2.08)
Yes	1	1	1	1	1	1	1	1
**PPE available**								
No	0.20 (0.11, 0.38)^*^	0.14 (0.06, 0.32)^*^	1.75 (1.12, 2.74)^*^	1.36 (0.61, 2.55)	1.07 (0.69, 1.64)	0.81 (0.46, 1.43)	1.88 (0.85, 4.18)	1.53 (0.74, 2.78)
Yes	1	1	1	1	1	1	1	1
**Behavioral response**								
Poor EBR	0.34 (0.22, 0.54)^*^	0.30 (0.16, 0.89)^*^	2.62 (1.71, 4.02)^*^	2.13 (1.18, 3.57)^*^	2.34 (1.56, 3.51)^*^	1.24 (1.85, 2.48)^*^	1.99 (1.06, 3.75)^*^	1.69 (1.02, 3.17)^*^
Good EBR	1	1	1	1	1	1	1	1
**Perception**								
Poor perception	3.57 (2.16, 5.89)^*^	3.27 (1.98, 4.47)^*^	1.93 (1.92, 4.12)	1.47 (1.88, 2.64)^*^	1.68 (1.09, 3.38)^*^	1.28 (1.09, 2.85)^*^	2.13 (1.60, 4.12)^*^	1.98 (1.56, 3.95)^*^
Good perception	1	1	1	1	1	1	1	1
**Experience COVID-19**								
No	0.89 (0.58, 1.37)	0.72 (0.43, 1.47)	0.61 (0.40, 0.94)^*^	0.51 (0.53, 1.58)	0.70 (0.47, 1.03)	0.61 (0.35, 1.29)	1.76 (0.92, 3.36)	1.62 (0.75, 2.12)
Yes	1	1	1	1	1	1	1	1
**Friend or families have COVID-19**								
No	0.65 (0.37, 1.12)	0.52 (0.28, 1.54)	0.45 (0.28, 0.72)^*^	0.35 (0.56, 1.51)	0.84 (0.52, 1.34)	0.71 (0.49, 1.29)	1.27 (0.57, 2.83)	0.98 (0.47, 1.95)
Yes	1	1	1	1	1	1	1	1
**COVID-19 in their settings**								
No	1.94 (1.27, 3.98)^*^	1.23 (0.58, 2.13)	0.71 (0.47, 1.08)	0.42 (0.35, 1.99)	0.39 (0.26, 0.58)^*^	0.29 (0.21, 1.69)	0.84 (0.45, 1.58)	0.75 (0.52, 1.45)
Yes	1	1	1	1	1	1	1	1

*PPE, personal protective equipment; EBR, early behavioral response; 1-reference*.

* *Statistically significant at p-value < 0.05 in bivariable and multivariable logistic regression*.

Female participants were two times more likely to have symptoms of depression than male participants [AOR = 1.86; 95 CI = (1.20, 3.56)]. Married participants were 2.87 times more likely to have symptoms of depression than single participants [AOR = 2.87; 95% CI = (2.03, 4.30)]. HCWs working in the emergency unit had higher odds of having symptoms of depression than HCWs who were working in the outpatient department [AOR = 2.11; 95% CI = (1.27, 4.61)]. HCWs who have working experience of <5 years were two times more likely to have symptoms of depression than HCWs who have working experience of more than 10 years [AOR = 2.07; 95% CI = (1.89, 4.84)]. HCWs who have poor behavioral responses toward the COVID-19 pandemic were two times more likely to have symptoms of depression compared to HCWs who have good behavioral responses [AOR = 2.13; 95% CI = (1.18, 3.57)]. The study participants who have poor perception toward COVID-19 were 1.47 times more likely to have symptoms of depression compared to those who have good perception toward the COVID-19 pandemic [AOR = 1.47; 95% CI = (1.88, 2.64)] (Table 5).

Female participants were two times more likely to have symptoms of anxiety than male participants [AOR = 1.90; 95% CI = (1.12, 3.24)]. HCWs working in the emergency unit were three times more likely to have symptoms of anxiety than HCWs working in the outpatient department [AOR = 2.79; 95% CI = (1.59, 4.89)]. HCWs working in the surgical unit were three times more likely to have symptoms of anxiety when compared with HCWs working in the outpatient department [AOR = 3.26; 95% CI = (1.53, 6.89)]. Poor behavioral response and poor perception toward the COVID-19 pandemic were also significantly associated with symptoms of anxiety (Table 5).

Female participants were 2.16 times more likely to have symptoms of insomnia than male participants [AOR = 2.16; 95% CI = (1.58, 4.38)]. Married participants were three times more likely to have symptoms of insomnia than single participants [AOR = 3.31; 95% CI = (1.56, 6.68)]. HCWs working in the emergency units were three times more likely to have symptoms of insomnia than HCWs working in the outpatient department [AOR = 2.74, 95% CI = (1.85, 6.45)]. HCWs who have working experience of <5 years were 2.45 times more likely to have symptoms of insomnia when compared with HCWs who have working experience >10 years [AOR = 2.45; 95% CI = (1.28, 4.90)]. HCWs who have poor early behavioral responses toward the COVID-19 pandemic were two times more likely to have symptoms of insomnia when compared with HCWs who have good behavioral responses toward the COVID-19 pandemic [AOR = 1.69; 95% CI = (1.02, 3.17)]. HCWs who have poor perception toward the COVID-19 pandemic were two times more likely to have symptoms of insomnia compared to HCWs who have good perception toward the COVID-19 pandemic [AOR = 1.98; 95% CI = (1.56, 3.95)] (Table 5).

## Discussion

The current finding indicated that self-reported psychological problems are prevalent in HCWs during the COVID-19 pandemic. Moreover, various HCWs experienced significant symptoms of psychological distress, depression, anxiety, and insomnia. We further identified the possible risk factors associated with symptoms of psychological distress, depression, anxiety, and insomnia, including socio-demographic characteristics, working area, early behavioral response, perception, training, and shortage of medical supply, and so on.

We found nearly three-fifths of the overall prevalence of psychological distress symptoms among HCWs. This result is consistent with studies reported in New York (8) and China (29) during the current COVID-19 pandemic. The symptoms of psychological distress in our finding is higher than the studies conducted in Singapore (30), in Singapore and India (17), in Iran (31), and in Italy (32). On the other hand, this prevalence is lower than the studies conducted in Pakistan, Turkey, and China, which found 72.4, 76.4, and 71.5% prevalence of psychological distress, respectively (5, 9, 12). The difference may be due to a difference in study participants and measurement tools. In addition, medical laboratory professionals, physicians, and nurses had a higher prevalence of psychological distress, which is higher than the study reported in China (9) and Turkey (12). Additionally, physicians and medical laboratory professionals were significantly associated with symptoms of psychological distress, which is in line with the studies done in China and Turkey (9, 12). HCWs are under high pressure during the current pandemic, because COVID-19 has led to high morbidity and mortality rates, overloaded intensive care units, and introduced fear and psychological distress in health care facilities (7).

In this study, we found 16.3% of HCWs reported moderate to severe symptoms of depression which were similar to findings in Hubei, China (33), and in line with the study conducted in Fujian, China (34). This finding is higher than the studies reported in India and Singapore (17) and Singapore (30). Conversely, this result is lower than the studies reported in Turkey (12), in Hong Kong, China (33), in Italy (11), in New York (8), in Nepal (35), and in Iran (31). This may be explained by the difference in data collection tools, cut-off points, and geographical location. Moreover, we found a higher rate of depression symptoms moderate to severe among laboratory professionals, physicians, and nurses, which was similar to the study conducted in China that revealed 11.2% of technicians, 12.9% of physicians, and 12.0% of nurses had symptoms of depression (29). Another study in China reported similar results that confirmed moderate to severe symptoms of depression for physicians and nurses (9). Additionally, this result is lower than the study result reported in Turkey that found 35.7% of physicians, 42.1% of nurses, and 37.1% of other HCWs had reported moderate to severe symptoms of depression (12).

In our finding 30.7% of HCWs had reported moderate to severe symptoms of anxiety, which is consistent with the studies reported in a systematic review and meta-analysis (36), in New York (8), in Iran (31), and in China (16). This result was higher than the studies conducted in Italy (11), in Singapore (30), in Singapore and India (17), and a systematic review and meta-analysis (37). In contrast, this rate was lower than studies reported in Nepal (35), in China (10), and Turkey (12). The difference may be due to the variation in assessment tools or the number of sample sizes. In addition to this, we found a higher prevalence of anxiety symptoms in a medical laboratory, physicians, and nurses. This prevalence is higher than the study done in China that found 14.5% of technicians, 12% of physicians, and 14.9% of nurses had reported moderate to severe symptoms of anxiety (29). Other studies in China and Turkey also found a low prevalence of anxiety symptoms among physicians, nurses, and other technicians who reported moderate to severe anxiety (9, 12).

During the COVID-19 pandemic, sleep disturbances among medical staff have been commonly reported and are major factors for lack of self-care and mood disturbance (38). This inadequate sleep can produce various health problems for HCWs and minimize the attention they give to their activities (39). We found that 15.9% of HCWs had reported moderate to severe symptoms of insomnia. Similarly, we found that medical laboratory professionals, physicians, and nurses had reported a higher prevalence of insomnia. This finding is higher than the studies reported in Italy (11). Conversely, this result is lower than the studies conducted in New York (8), Turkey (12), and China (10). The difference may be explained by the difference in tools and their cut-off points and the difference affected by COVID-19 incidence.

Perception is vital to determining defensive health behaviors and is used for the response and organization of individual insight to safety measures (40, 41). In our finding, 51.8% of HCWs had a good perception of COVID-19 infection. More than two-fifth of the nurses had reported poor perception toward the COVID-19 pandemic. Quite the opposite, 61.2% of physicians and three-fourths of medical laboratory professionals had reported good perception toward COVID-19 pandemic prevention. HCWs who had poor perception were significantly associated with psychological distress, depression, anxiety, and insomnia. This finding is lower than the study result reported in Saudi Arabia (42). This may be due to the poor perception influencing HCWs' low use of standard measures to prevent COVID-19 infection while good perception is a good indicator for the prevention of the pandemic.

Early behavioral response and the types of prevention mechanism used might be useful for developing coping behaviors. Studies revealed that early behavioral response to the COVID-19 pandemic is important to reduce transmission of the infection, stress reduction, and develop better-coping behaviors toward the pandemic (8, 43, 44). In our study, 60.7% of HCWs had good early behavioral responses to the COVID-19 pandemic. Of these, nearly three-fifths of the nurses, physicians, and medical laboratory professionals had reported good early behavioral responses toward the COVID-19 pandemic. Similarly, HCWs who had inadequate early behavioral response to the COVID-19 pandemic were significantly associated with psychological distress, depression, anxiety, and insomnia, which is in line with the study result in New York (8). HCWs that have inadequate early behavioral responses have a greater fear of the pandemic (45). An inadequate early behavioral response can be associated with a lack of adequate facemasks, sanitizer, and maintaining social distancing (46).

We found that female participants had significantly higher odds of having symptoms of psychological distress, depression, anxiety, and insomnia than male participants. This result was similar to the study results in Wuhan that reported 14.2, 25.2, and 31.6% of female participants had symptoms of depression, anxiety, and acute stress, respectively (47). Similarly, Turkey's study found consistent results that female participants were significantly associated with depression, anxiety, and psychological distress (12). Studies found that being female related to high-risk factors for mental disorders, and was more statistically significant to secondary traumatization during COVID-19 pandemic (36, 48). There is also a gender difference in family responsibility, more occupational exhaustion, and inequality in domestic labor that leads to mental disorders (49). These factors may enhance women's high susceptible to mental impact during the COVID-19 pandemic crisis.

Our study found that married participants had significantly higher odds of having psychological distress, depression, anxiety, and insomnia than single participants. This result is similar to the study result reported in China (50). HCWs who have a diploma and bachelor's degree were significantly associated with symptoms of psychological distress. This result contradicts the study conducted in Wuhan, China that found higher education attainment was associated with elevated psychological symptoms (51). This difference may be due to the category of educational level.

In our study, HCWs working in the emergency, surgical, and medical unit had higher significant symptoms of psychological distress, depression, anxiety, and insomnia. This finding is similar to the result reported in Fujian Province, China that found working in emergency, infectious disease departments, and the ICU made participants twice as likely to develop symptoms of mental illness (34). Working in high-risk exposure areas creates a high psychological burden for frontline HCWs, and leads to the higher rates of psychological distress, depression, anxiety, and insomnia (12). Additionally, HCWs who have working experience of <5 years had higher significant odds of having depression and insomnia. This finding is in line with studies' findings in Turkey and Wuhan (12, 47). Studies documented that HCWs with less experience might have a higher fear of severe infection and have low coping techniques, and therefore develop a higher level of depression and insomnia during a disease outbreak (34, 50).

We found that HCWs who have not been trained about COVID-19 infection prevention and control were significantly associated with symptoms of psychological distress. Additionally, HCWs who have a shortage of personal protective equipment were significantly related to symptoms of psychological distress. Lack of training or up-to-date information about COVID-19 infection and shortage of protective medical equipment may provoke psychological distress. In contrast, the study found that having adequate personal protective equipment or medical resources, up-to-date and correct health care information, and taking preventive measures were protective factors from psychological disorders during the current COVID-19 pandemic (36).

Finally, as a limitation, this study does not show a causal relationship due to its cross-sectional nature. The study has been conducted at healthcare facilities located in the North Shoa Zone of the Oromiya region, hence, the findings cannot be generalizable to the other Ethiopian regions.

## Conclusion

Health care workers in Ethiopia had experienced a high level of psychological distress, depression, anxiety, and insomnia. Physicians, nurses, and medical laboratory professionals had a higher prevalence of moderate to severe symptoms of psychological distress, depression, anxiety, and insomnia. Female and married participants, those working in the emergency unit, those with inadequate early behavioral responses, and those with a poor perception of the COVID-19 pandemic were identified as being significantly more at risk for psychological distress, depression, anxiety, and insomnia. The Ethiopian Federal Ministry of Health should incorporate psychological support for HCWs during the COVID-19 pandemic.

## Data Availability Statement

The raw data supporting the conclusions of this article will be made available by the authors, without undue reservation.

## Ethics Statement

The studies involving human participants were reviewed and approved by Salale University Ethical Review Committee. The patients/participants provided their written informed consent to participate in this study.

## Author Contributions

KJ conceived the study, involved in the study design, reviewed the article, analyzed, report writing, drafted, and revised the manuscript. BD and TG contributed to data analysis, report writing, drafted the manuscript, and gave final approval of the version to be published. All authors agree to be accountable for all aspects of the work.

## Conflict of Interest

The authors declare that the research was conducted in the absence of any commercial or financial relationships that could be construed as a potential conflict of interest.

## Abbreviations

AOR: Adjusted Odds Ratio
CI: Confidence Interval
COVID-19: Corona Virus Disease-2019
GAD-7: General Anxiety Disorder-7
HCWs: health care workers
IES-R: Impact of Event Scale-Revised
ICU: Intensive care unit
ISI: Insomnia Severity Index
PHQ-9: Patient Health Questionnaire-9
SPSS: Statistical Package for Social Science
WHO: World Health Organization.
